# Aging Lung: Molecular Drivers and Impact on Respiratory Diseases—A Narrative Clinical Review

**DOI:** 10.3390/antiox13121480

**Published:** 2024-12-02

**Authors:** Paweł Górski, Adam J. Białas, Wojciech J. Piotrowski

**Affiliations:** 1 Department of Pneumology, Medical University of Lodz, 90-419 Lodz, Poland; adam.bialas@umed.lodz.pl (A.J.B.); wojciech.piotrowski@umed.lodz.pl (W.J.P.); 2 Department of Pulmonary Rehabilitation, Regional Medical Center for Lung Diseases and Rehabilitation, Blessed Rafal Chylinski Memorial Hospital for Lung Diseases, 91-520 Lodz, Poland

**Keywords:** aging, oxidative stress, cellular senescence, COPD, IPF, asthma, fibrotic ILD

## Abstract

The aging process significantly impacts lung physiology and is a major risk factor for chronic respiratory diseases, including chronic obstructive pulmonary disease (COPD), idiopathic pulmonary fibrosis (IPF), asthma, and non-IPF interstitial lung fibrosis. This narrative clinical review explores the molecular and biochemical hallmarks of aging, such as oxidative stress, telomere attrition, genomic instability, epigenetic modifications, proteostasis loss, and impaired macroautophagy, and their roles in lung senescence. Central to this process are senescent cells, which, through the senescence-associated secretory phenotype (SASP), contribute to chronic inflammation and tissue dysfunction. The review highlights parallels between lung aging and pathophysiological changes in respiratory diseases, emphasizing the role of cellular senescence in disease onset and progression. Despite promising research into modulating aging pathways with interventions like caloric restriction, mTOR inhibitors, and SIRT1 activators, clinical evidence for efficacy in reversing or preventing age-related lung diseases remains limited. Understanding the interplay between aging-related mechanisms and environmental factors, such as smoking and pollution, is critical for developing targeted therapies. This review underscores the need for future studies focusing on therapeutic strategies to mitigate aging’s detrimental effects on lung health and improve outcomes for patients with chronic respiratory conditions.

## 1. Introduction

Aging is a complex, multifactorial process that involves physiological, molecular, and structural changes across various organ systems, including the lungs.

At the molecular level, oxidative stress plays a critical role in the progression of aging. Oxidative stress refers to an imbalance between the production of reactive oxygen species (ROS) and the body’s ability to neutralize them through antioxidant defenses [1]. ROS are highly reactive molecules primarily generated in the mitochondria, and they may interact with proteins, lipids, and nucleic acids, causing cumulative cellular damage. This process becomes increasingly significant with age as mitochondrial function declines, leading to the accumulation of damaged mitochondria and increased ROS production [2].

In the aging lung, oxidative stress is a central driver of cellular senescence. Cellular senescence serves protective functions in youth, such as preventing the proliferation of damaged cells and aiding in tissue repair. However, with age, the accumulation of senescent cells and their secretion of pro-inflammatory mediators (senescence-associated secretory phenotype or SASP) foster chronic low-grade inflammation [3,4]. ROS-induced damage to DNA, proteins, and lipids exacerbates cellular dysfunction and accelerates senescence, further perpetuating a vicious cycle of inflammation and oxidative stress. Additionally, oxidative stress contributes to the deterioration of proteostasis, a crucial process for maintaining protein synthesis and degradation. Impaired proteostasis exacerbates age-related diseases and hinders tissue repair, further weakening lung function [5].

Moreover, oxidative stress plays a central role in mitochondrial dysfunction, one of the key antagonistic hallmarks of aging. Mitochondria are the primary source of ROS generation, and as mitochondrial DNA (mtDNA) is particularly susceptible to oxidative damage, this can lead to progressive mtDNA mutations over time. Damaged mitochondria become less efficient at producing energy and more prone to generating excessive ROS [6], thus promoting further cellular damage and senescence. This process, termed mitohormesis, can promote longevity in youth by activating stress response pathways, but in aged tissues, the accumulation of dysfunctional mitochondria contributes to the onset of age-related diseases [7].

The aim of this narrative review was to provide an in-depth exploration of the physiology of aging, with a focus on key molecular and biochemical processes, including oxidative stress, telomere attrition, genomic instability, epigenetic modifications, loss of proteostasis, and impaired macroautophagy. Additionally, we examined the role of aging in the development and progression of lung diseases where scientific evidence supports its involvement, specifically COPD, asthma, IPF, and non-IPF interstitial lung fibrosis.

A search of the PubMed database was conducted to identify relevant articles addressing lung senescence and its role in lung diseases. The search included articles published up to 30th of September 2024, and utilized the following search string: (“lung senescence”[Title/Abstract] OR “pulmonary senescence”[Title/Abstract] OR “cellular senescence”[Title/Abstract]) AND (“lung diseases”[MeSH] OR “COPD”[Title/Abstract] OR “asthma”[Title/Abstract] OR “pulmonary fibrosis”[Title/Abstract] OR “lung fibrosis”[Title/Abstract] OR “interstitial lung disease”[Title/Abstract]) AND (“aging”[MeSH] OR “aging”[Title/Abstract] OR “age-related”[Title/Abstract]). Articles were included if they discussed cellular or tissue senescence in the lungs, examined the relationship between senescence and lung diseases such as COPD, asthma, idiopathic pulmonary fibrosis, or non-IPF interstitial lung fibrosis, were published in English, and included original research, reviews, or meta-analyses. The reference sections of included manuscripts were also reviewed to identify additional relevant studies.

## 2. Lung Structure and Physiology in Aging

**Figure 1:**
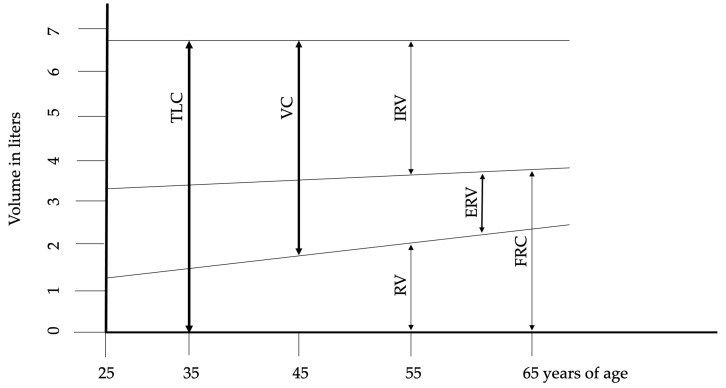
Changes of lung volumes during aging. Abbreviations: ERV—expiratory reserve volume, FRC—functional residual capacity, IRV—inspiratory reserve volume, RV—residual volume, TLC—total lung capacity, VC—vital capacity. Adopted from Janssens et al. [9].

The maximum number of alveoli is reached by approximately 12 years of age, correlating with an increase in respiratory function that peaks around age 20 in females and 25 in males. The most significant physiological changes associated with aging include reduced static elastic recoil, decreased chest wall compliance, and weakened respiratory muscles. These phenomena are the basis for almost all other age-related changes in lung function, including alterations in chest wall mechanics, respiratory muscle function, and lung tissue structure. described the fundamental structural and physiological alterations in respiratory function associated with aging. The reduction in chest wall compliance appears to be linked to calcification and osteoporosis across bone structures and joints, contributing to increased dorsal kyphosis and an expansion in anteroposterior chest diameter. These changes also impact the diaphragm, reducing its contractile strength. Functional residual capacity (FRC) rises in conjunction with the age-related decline in muscle mass and strength. Impaired nutritional status, common in elderly individuals, exacerbates respiratory muscle weakness, particularly through a reduction in muscle fiber number. Subclinical cardiac insufficiency further compromises blood flow and oxygen delivery to muscles. Additionally, alterations in the cardiovascular system, coupled with subclinical systemic inflammation, negatively affect muscle function. Although changes in lung volumes are consistently reported, the onset and progression of these changes vary among individuals. Figure 1 illustrates the changes in lung volumes. After the age of 50, progressive structural changes in the lungs become apparent. The smaller airways begin to enlarge due to the expansion of the collagen fiber network surrounding the alveolar ducts. The uniform enlargement of the alveolar spaces is associated with a reduction in alveolar surface tension and compliance. Despite these changes, the functional properties of airway smooth muscle generally remain stable or exhibit only a slight decline compared to younger individuals. Senile emphysema, or age-related emphysema, is distinguished by the absence of alveolar destruction [8,9,10,11].

## 3. Molecular and Biochemical Hallmarks of Aging

López-Otín and colleagues identified nine hallmarks of aging, categorized into three groups: primary, antagonistic, and integrative. The primary hallmarks include telomere attrition, genomic instability, epigenetic alterations, loss of proteostasis, and impaired macroautophagy [12]. Telomeres, consisting of a repetitive nucleotide sequence (TTAGGG) of complementary double-stranded DNA, progressively shorten with each cell replication cycle [13]. They play a crucial role in protecting chromosome ends and maintaining DNA stability. In the absence of telomeres, DNA damage ensues. Telomeres also have a protective role in cancer prevention and contribute to the deceleration of the aging process. However, telomere attrition, though considered a genomic safeguard by limiting uncontrolled cell proliferation, may have adverse effects by impairing tissue regeneration and inhibiting cell division [14]. Genomic instability, characterized by cumulative damage to the genome over time, plays a critical role in the aging process [15].

Epigenetic alterations result in modifications to gene expression. The dysregulation of transcription, driven by histone acetylation, histone modification, heterochromatin loss or gain, and changes in DNA methylation, contributes to the aging process [16]. Loss of proteostasis refers to the decline in protein synthesis, dysfunction, or degradation. The breakdown of proteostasis is a significant factor in aging and is implicated in numerous age-related diseases [5].

Three key factors are classified as antagonistic hallmarks: deregulated nutrient sensing, mitochondrial dysfunction, and cellular senescence. Caloric restriction, while maintaining adequate nutritional quality, has been shown to extend lifespan in *C. elegans* and rodent models. Inhibition of mTORC1 (mammalian target of rapamycin complex 1), a protein complex that regulates protein synthesis and cellular metabolism, has also been demonstrated to prolong lifespan [17,18]. Mitochondria generate the majority of ROS, which interact with various biomolecules, including nucleic acids, phospholipids, and proteins. While proteins and phospholipids can be replaced over time, DNA damage is cumulative. Mitochondrial function declines with age, leading to the accumulation of damaged mitochondria and increased ROS production, ultimately resulting in cell death [2]. During youth, this process occurs as an adaptive response to stress, termed mitohormesis, which promotes longevity [12]. Mitochondrial DNA (mtDNA) is particularly vulnerable to ROS, and some researchers have suggested that aging is associated with progressive mtDNA mutations. Mitochondrial dysfunction contributes to cellular senescence through various mechanisms, including mitochondrial genomic instability, involvement in inflammatory processes, disruption of proteostasis, dysregulation of nuclear gene expression, and the production of mitochondrial-derived peptides [7].

Cellular senescence serves a protective role against cancer and aids in wound healing during youth. However, in adulthood, it is associated with a reduced lifespan and the onset of age-related diseases (see Table 1).

The integrative hallmarks of aging include stem cell exhaustion, alterations in intracellular communication, chronic inflammation, and dysbiosis. Stem cell exhaustion is a well-established contributor to aging, leading to diminished tissue regenerative capacity [19]. Intracellular communication, coordinated by complex molecular and biochemical networks, ensures homeostasis and plays a crucial role in regulating lifespan. As these networks progressively deteriorate with age, they contribute to reduced lifespan and heightened susceptibility to age-related diseases [20].

Autophagy refers to the lysosomal degradation and recycling of intracellular components, which is essential for maintaining proteostasis. The impairment of macroautophagy is a central feature of the aging process and is considered one of its hallmarks [21].

Chronic, low-grade inflammation, even at a subclinical level, is a persistent characteristic of aging. Increased levels of interleukin-6 (IL-6) and interleukin-8 (IL-8) in bronchoalveolar lavage (BAL) fluid are frequently observed in the elderly [22]. While cellular senescence plays a protective role in preventing malignant transformations by eliminating potentially cancerous cells, the accumulation of senescent cells over time can promote disease development [23]. Senescent cells secrete an array of inflammatory mediators, including cytokines and other bioactive proteins, in a process known as the SASP. The number of senescent cells and the expression of SASP increase with age. SASP contributes to autocrine signaling in the lungs, promoting wound repair by recruiting inflammatory cells to clear senescent cells. However, the removal of these cells is a slow process, leading to their accumulation, often accompanied by elevated levels of specific interleukins [24,25]. The buildup of senescent cells and SASP promotes chronic inflammation, frequently observed in older individuals without clinically diagnosed diseases [3,4]. Cellular senescence in lung resident cells is a hallmark of normal aging, but senescence in circulating immune cells also plays a significant role in the aging process, contributing to the progression of age-related diseases.

## 4. From Senescence to Aging Lung

**Figure 2:**
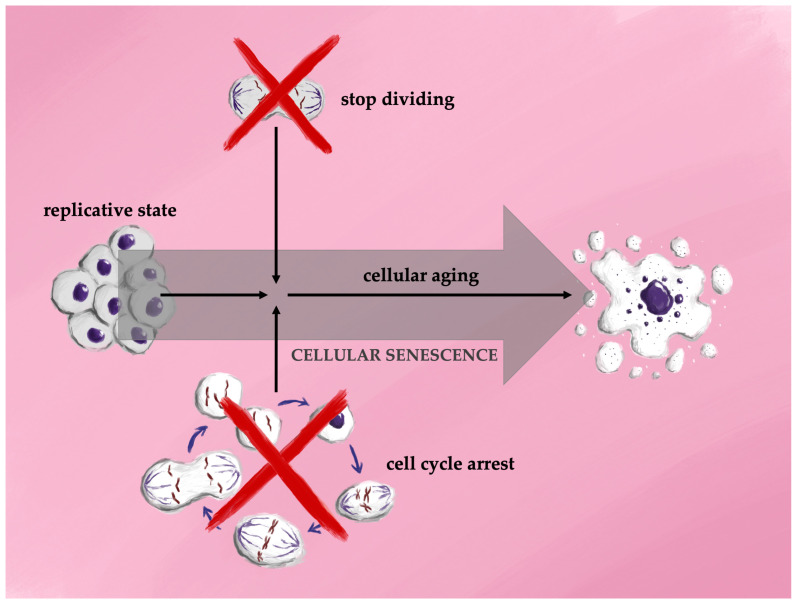
Schematic representation of the different stages of cellular life.

Cellular senescence is a process in which a cell undergoes irreversible cell cycle arrest, accompanied by structural changes, secretion of bioactive molecules, and various epigenetic modifications. In long-term cultures of proliferating cells, primary cells or stem cells cease division after a finite number of replications, a phenomenon known as the Hayflick limit, followed by a state of cell cycle arrest termed replicative senescence. Although the mechanisms underlying the Hayflick limit are not fully elucidated, factors such as telomere attrition and genomic instability are thought to play significant roles in driving replicative senescence [26]. The first association between cellular senescence and aging was reported in 1961, when Hayflick and Moorhead demonstrated that human fetal cells could be cultured for longer durations before reaching replicative limits compared to adult human cells [27]. In 2012, Longo et al. introduced distinctions between two forms of cellular lifespan: cellular aging, which describes the gradual functional decline leading to increased susceptibility to cell death, and replicative aging, which refers to the phase where dividing cells undergo permanent cell cycle arrest in preparation for senescence. This phase is characterized by an inflammatory state, decreased cell proliferation, and eventual prolonged survival before the cell’s ultimate death [28] (Figure 2).

### 4.1. The Mechanisms of Cellular Senescence

**Figure 3:**
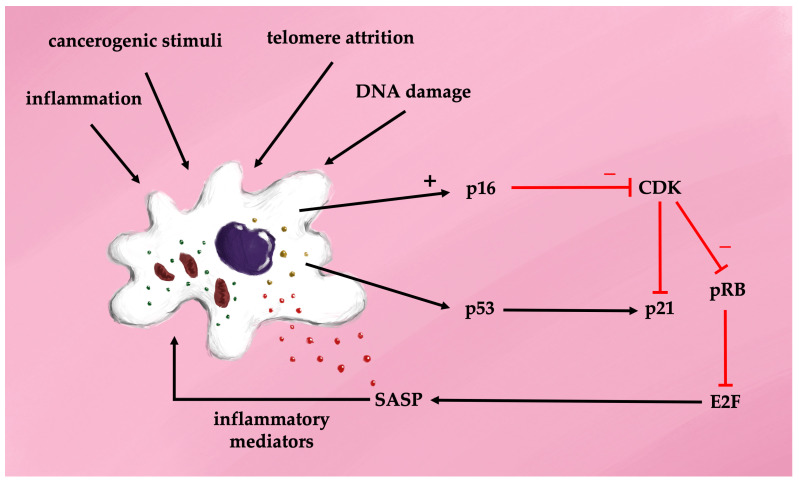
Cell transformation in senescence—the senescence-associated secretory phenotype (SASP).

The cell cycle depends on passing through four checkpoints: the G1-S transition, the S phase, the G2 to M transition, and the mitotic spindle checkpoint. The G1-S transition is regulated by the p16-Rb (retinoblastoma) pathway, where the Rb protein family inhibits the transcription factor E2F1, leading to cell cycle arrest. Senescent cells continue protein synthesis via mTOR activation [29]. Morphologically, senescent cells are characterized by a flattened, enlarged shape, telomere attrition, and overexpression of cyclin-dependent kinase inhibitors p16 and p21, as well as increased senescence-associated beta-galactosidase (SA-beta-gal) activity. Interestingly, mice lacking p16-positive cells exhibit extended lifespans [30]. The mechanisms underlying cellular aging pathways involve a sequence of overlapping events triggered by diverse stimuli, including ROS, endoplasmic reticulum (ER) stress, mitochondrial dysfunction, epigenetic modifications, DNA damage, oncogene activation, and other stressors [31,32,33]. In naïve cells exposed to these stimuli, intracellular pathways drive proliferation, apoptosis, or resistance to autophagy. Chronic oxidative stress is central to the pathogenesis of senescent cells, primarily through the activation of p53 and p16 proteins, which inhibit cyclin-dependent kinases, resulting in G1 cell cycle arrest [34]. Two major signaling pathways drive cellular senescence during aging. The first relies on the p16-retinoblastoma (Rb) pathway, where p16 competes with cyclin-dependent kinases 4/6 (CDK4/6) to reduce Rb phosphorylation and prevent transcription factor activation. The second pathway involves p53 and cyclin-dependent kinase inhibitor 1 (p21). In response to stress, the tumor suppressor protein p53 is activated, leading to upregulation of p21 and inhibition of Rb. Both pathways ultimately result in cell cycle arrest [35] (Figure 3). It was discovered over 20 years ago that progressive telomere shortening activates suppressor pathways, including p53 phosphorylation and elevated p21 production, leading to cellular senescence [36].

The activation of the SASP is primarily regulated by nuclear factor kappaB (NF-kappa B), a key factor in various inflammatory signaling pathways [37]. NF-kappaB signaling occurs through two principal pathways: the canonical and non-canonical pathways. The canonical pathway involves NF-kappaB essential modulator (NEMO) and is activated by TNF-alpha, interleukin-1 beta, or lipopolysaccharides (LPS), leading to phosphorylation of the inhibitor of kappa B (I-kappaB) kinase (IKK). This phosphorylation results in the proteasomal degradation of I-kappaB proteins and translocation of the NF-kappaB complex (p50/p65) into the nucleus. The non-canonical pathway, in contrast, is independent of NEMO and is activated via membrane-bound receptors, allowing the NF-kappaB complex to translocate into the nucleus without I-kappaB involvement. ROS play a significant role in inducing cellular senescence through NF-kappaB signaling [38]. Both canonical and non-canonical NF-kappaB pathways are activated by ROS and DNA damage. NF-kappaB helps maintain cellular senescence by promoting DNA repair and ensuring genome stability. Its activity increases with age, and inhibition of NF-kappaB has been shown to delay DNA damage-induced senescence, reduce SASP production, and delay the onset of age-related diseases [39]. Once activated, NF-kappaB triggers self-sustaining mechanisms through the release of cytokines that further activate NF-kappaB, although these processes can potentially be halted or reversed [40,41]. Therefore, NF-kappaB might be a key factor in therapeutic interventions in the treatment of diseases associated with accelerated aging.

Several NF-kappaB inhibitors have been identified. Some pharmaceuticals, primarily used in anticancer therapy, have previously unrecognized effects on NF-kappaB signaling and might be considered for managing accelerated aging [42].

**Figure 4:**
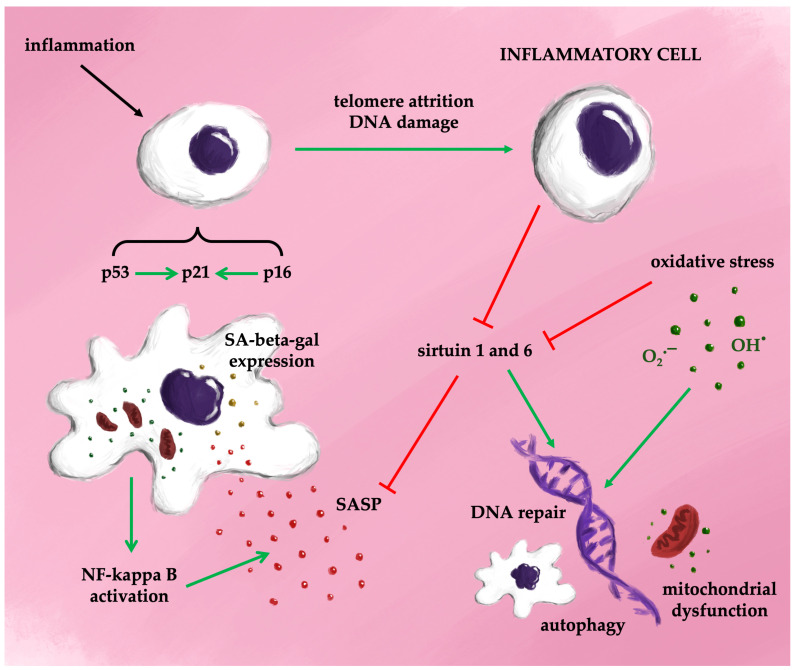
Possible mechanisms of senescence (simplified).

Figure 4 provides a simplified overview of the potential mechanisms driving cellular senescence.

### 4.2. Senescence Not Only in Inflammation and Aging

If senescence is an important part of aging, it raises the question of whether it is also of real importance as a normal part of lung development. Although studies in mice have shown that it contributes to the development of the neural tube, pharynx, hind limb, and gut, whether it plays a role in the lung parenchyma in embryos is not yet known [43,44]. However, certain indirect pieces of evidence suggest that senescence plays a role in the development and maturation of the lungs [43]. Pathological pregnancies undoubtedly have an influence on lung development, and there is substantial data showing an indirect effect on aging in lung tissue during prenatal development [45]. Several factors during delivery and immediately afterwards could potentially trigger perinatal aging. The fetus grows in an environment with limited oxygen, and this low oxygen level promotes the development of the lungs through branching morphogenesis [46,47]. Oxygen supplementation in neonates leads to abnormal breathing test results in children at 8 years old [48]. The most likely explanation for the results of this observation is that oxygen usage in newborns leads to fibroblast aging. It was observed that fibroblasts exposed to prolonged oxygen exposure show signs of aging [49]. Many authors have observed dysregulation of neonatal growth alongside abnormal lung branching and vascular morphogenesis, potentially due to fibroblasts’ senescence [50,51]. Furthermore, the activation of senescence in an oxygenated environment in infants during intensive therapy may result in bronchial asthma and lung fibrosis. This was demonstrated in a model of isolated fetal smooth muscle [52]. Therefore, senescence appears to be a significant factor in lung disorders in children. There is no information available on senescence in human adolescents. Research on aging tends to be concentrated on individuals aged 50 and older.

### 4.3. Markers of the Cellular Senescence

Senescent cells actively produce and secrete a wide array of inflammatory mediators, including cytokines and other biologically active proteins. Inflammation-induced cellular senescence fosters a self-perpetuating cycle, wherein inflammation both triggers and sustains the senescent state. Notably, interleukin-6 (IL-6) and interleukin-8 (IL-8), key components of the SASP, play crucial roles in this process through autocrine signaling, further promoting cellular senescence [41]. While in vitro studies have unequivocally demonstrated the presence of senescent cells, their definitive identification in vivo remains elusive. The use of senescence biomarkers offers a potential avenue to address this gap. Among the most reliable histological biomarkers are senescence-associated beta-galactosidase (SA-β-Gal) and lipofuscin [53]. Growth and differentiation factor 15 (GDF15), a member of the TGF-beta superfamily, is emerging as a potential plasma marker. Elevated GDF15 levels have been correlated with specific cardiovascular and respiratory symptoms associated with aging. Additionally, GDF15 has been detected in both tissue and bronchoalveolar lavage samples from individuals with IPF, as well as in murine models following bleomycin exposure [54,55,56].

However, there is consensus that no single biomarker currently offers sufficient specificity for the unequivocal identification of cellular senescence. Only a combination of markers can effectively identify senescent cells [57]. This lack of a unique and reliable biomarker may be attributable to the varying degrees of senescence that cells undergo in vivo over time. Some researchers suggest that the bystander effect further complicates the interpretation of study results. Consequently, multiple complementary markers are needed to identify senescent cells reliably. Techniques commonly used to detect senescence in vivo, such as flow cytometry, immunohistochemistry, immunofluorescence, and real-time PCR, typically focus on a single marker, which limits the ability to compare experimental outcomes. Furthermore, researchers cannot be certain that the markers being investigated are exclusive to senescence, as they are also present during advanced aging [26].

### 4.4. Telomeres in Aging

Telomeres are specialized DNA–protein complexes that protect the terminal regions of chromosomes, safeguarding genomic integrity. With each successive cell division, telomeres undergo incomplete replication, ultimately reaching the Hayflick limit, at which point their protective capacity diminishes, leading to DNA vulnerability and the onset of cellular senescence [58]. Multiple factors contribute to telomere shortening, including replication-dependent erosion, which induces chromosomal instability, activation of the DNA damage response (DDR), and cell cycle arrest upon reaching a critically shortened length [59,60]. While the DDR can be triggered by various stimuli, independent of telomere length, it can be reversed in proliferating cells, in contrast to cells arrested in the cell cycle [61].

Telomere attrition is a well-established biomarker of aging, though its role as a singular indicator should be approached with caution. There is considerable interest in exploring the potential for lifespan extension through telomere lengthening [62]. However, individuals with genetically longer telomeres in white blood cells have been shown to possess an increased risk for developing a range of cancers, including multiple myeloma, thyroid cancer, kidney cancer, melanoma, soft tissue sarcoma, breast cancer, non-Hodgkin’s lymphoma, bladder cancer, prostate cancer, and endometrial cancer [63]. The relationship between telomere length and cancer risk is further complicated by findings in lung cancer, where both shortened and elongated telomeres are implicated. In small cell lung cancer (SCLC), particularly among heavy smokers or women over the age of 65, shorter leukocyte telomere length is associated with an increased risk of mortality. In the Toronto Study, survival outcomes were examined in 823 lung cancer patients using blood leukocyte samples collected six months after diagnosis. Short telomeres (<10th percentile) were linked to poor prognosis in non-small cell lung cancer and adenocarcinoma. Furthermore, short telomeres were associated with increased mortality, even among never-smokers with lung cancer [64].

Although shortened telomeres are commonly associated with aging and a variety of chronic diseases, their use as the sole biomarker for senescence is insufficient. Telomere attrition is influenced by several mechanisms, with progressive replication-dependent shortening playing a critical role in promoting chromosomal instability, activating the DNA damage response, and initiating cell cycle arrest upon reaching a critical threshold [59,60]. Thus, while telomere length serves as a valuable marker in studies of cellular aging and senescence, it must be considered in conjunction with other markers to provide a comprehensive assessment.

### 4.5. Aging and Lung Disease

Multiple lung defense mechanisms decline in effectiveness with advancing age, contributing to increased susceptibility to respiratory disorders. Age-related cellular changes, diminished antioxidant capacity, and damage to the bronchial epithelium are closely associated with alterations in both local and systemic immune responses. Chronic conditions such as COPD and IPF are well-established age-related diseases of the respiratory system. Additionally, aging is a significant risk factor for acute respiratory illnesses, including pneumonia and acute respiratory distress syndrome (ARDS), and plays a pivotal role in the progression of chronic respiratory diseases such as asthma.

Ongoing research is focused on the underlying mechanisms of aging-induced lung remodeling, particularly the processes of extracellular matrix (ECM) deposition and fibroblast proliferation and activation. A central mechanism appears to involve cellular stress, which leads to mitochondrial dysfunction. This mitochondrial dysfunction is characterized by mutations in mitochondrial DNA (mtDNA), dysregulation of mitochondrial fusion and fission processes, and impaired mitochondrial respiration, all of which contribute to the pathophysiology of age-related respiratory diseases [65,66,67,68].

#### 4.5.1. COPD

In individuals over the age of 65, the risk of developing COPD is approximately five times higher than in those under 40 [69]. Notably, there are significant physiological and structural similarities between the aging lung and the pathophysiological changes observed in COPD. Both conditions are characterized by diminished lung function, reduced elastic recoil, airspace enlargement, and increased end-expiratory volume. Aging itself may act as a contributing factor in the progression of COPD [70]. Alveolar wall thickening is observed in normal aging without accompanying inflammation or fibrosis, whereas in COPD, the destruction of alveolar walls is a defining feature. Additionally, COPD is marked by airway inflammation, remodeling, and excessive mucus production, which result in irreversible airway obstruction. The accumulation of extracellular matrix components such as collagen, laminin, and fibronectin is typical in COPD but absent in the normal aging lung. Cellular senescence in epithelial, endothelial, fibroblast, and immune cells can be driven by environmental factors, such as tobacco smoke [71,72,73].

Aging is often conceptualized as tissue degeneration resulting from a persistent imbalance between pro-inflammatory factors and the body’s anti-inflammatory defenses, or between oxidative and antioxidative processes. “Inflammaging” refers to the chronic low-grade inflammation almost always developing during aging. It is a key contributor to both aging and the development of COPD. With advancing age, immune cells and humoral factors undergo changes that increase susceptibility to external irritants, including smoke. Numerous studies highlight interleukin-6 (IL-6) as a pivotal cytokine that accelerates both aging and the early onset of COPD [74,75]. COPD is a heterogeneous, progressive condition characterized by systemic inflammation, although the precise mechanisms driving the disease remain unclear. Emerging evidence points to unresolved endoplasmic reticulum (ER) stress as a potential initiator of COPD pathogenesis. ER stress at the molecular level promotes autophagy and cellular senescence, both of which are implicated in COPD progression [76]. Stem cell exhaustion is also a hallmark of aging, and in COPD, this manifests as a reduced capacity for stem cell proliferation and a depletion of multipotent basal progenitor cells early in the disease course [77].

Under normal physiological conditions, the endoplasmic reticulum (ER) is responsible for the synthesis and proper folding of cellular proteins. However, during ER stress, unfolded and misfolded proteins accumulate, activating the unfolded protein response (UPR). Although the UPR seeks to restore ER homeostasis by degrading these misfolded proteins, prolonged ER stress leads to cellular dysfunction and death [78,79,80]. Prolonged ER stress triggers inflammatory responses, autophagy, and apoptosis, with NF-kappa B playing a central role in the inflammatory cascade, which may also involve inflammasome activation. While UPR and autophagy typically help maintain cellular homeostasis, their overactivation can result in cell death [81].

Exposure to cigarette smoke induces the expression of cyclin-dependent kinase inhibitors p16, p19, and p21—markers of cellular senescence [82,83]. Tobacco smoke also causes oxidative stress in cells, leading to DNA damage, telomere shortening, and alterations in mitochondrial function. These oxidative changes promote cellular aging [84,85]. Several aging-related signaling pathways, including NF-kappa B, play central roles in COPD by regulating genes associated with cytokine production, adhesion molecules, metalloproteinases, and angiogenesis [86]. Sirtuin 1 (SIRT1) has been identified as a key factor in inhibiting aging [87]. The activation of the mammalian target of rapamycin (mTOR), which regulates many biological processes in aging, inhibits SIRT1 in individuals exposed to smoke, thereby affecting NF-kappa B expression [88]. Increased expression of p38 MAPK (mitogen-activated protein kinase) is also observed in the lung epithelial cells of COPD patients [89]. Several SIRT1 activators have been used against the acceleration of aging [90]. Resveratrol, a potent and specific SIRT1 activator, might be an interesting therapeutic candidate to counteract lung and muscle impairment in COPD. However, there is no convincing evidence that it influences life longevity or cardiovascular risk in COPD patients [91].

Savale et al. measured telomere length using quantitative polymerase chain reaction in leukocytes from 136 individuals with COPD, 113 smokers, and 42 nonsmokers. The median telomere length was 0.57 (0.23–1.18) in COPD patients, 0.79 (0.34–1.58) in smokers, and 0.85 (0.38–1.55) in nonsmokers. These findings indicate that telomere length in COPD patients was shorter than in healthy individuals [92]. Telomere shortening is correlated with the concentration of C-reactive protein, white blood cell count, and blood neutrophil count, as inflammatory markers [92,93,94]. Zhang et al. have identified six upregulated critical aging-related genes in COPD, two of which (NKG7, CKLF) showed a significantly negative association with lung function. NKG7, expressed in activated T and NK cells, plays a critical role in immune-related diseases, while CKLF contributes to immune and inflammatory responses [95]. These aging-related genes warrant further investigation as potential therapeutic targets for COPD prevention and treatment in the aging population.

#### 4.5.2. Senescence and Asthma

The role of senescence in the pathophysiology of asthma, particularly in older adults, remains unclear. However, senescence markers such as p16 and p21 are expressed at higher levels in asthmatic individuals compared to healthy controls. Thymic stromal lymphopoietin (TSLP), a major mediator of asthmatic inflammation, induces the production of p16 and p21 in a dose-dependent manner. In mice, blocking TSLP with a STAT3 inhibitor has been shown to prevent epithelial senescence, airway remodeling, and the expression of p16 and p21 [96]. Lung fibroblasts from mild asthmatics also exhibit higher p21 expression [97].

#### 4.5.3. Idiopathic Pulmonary Fibrosis

In approximately two-thirds of patients with IPF, disease onset occurs after the age of 60. IPF is a chronic, progressive interstitial lung disease of unknown etiology, characterized by the excessive accumulation of fibroblasts, myofibroblasts, alveolar epithelial cells (AECs), macrophages, and a collagen-rich ECM. Impaired apoptosis of myofibroblasts is a key pathogenic mechanism driving the fibrotic process [98]. Alveolar epithelial type II (ATII) cells play a critical role in the early stages of IPF, with repeated injury to ATII cells leading to dysregulated fibrotic repair. Aging exacerbates this process by inducing endoplasmic reticulum (ER) stress, reducing autophagy, shortening telomeres, impairing mitochondrial function, and altering epigenetic regulation in ATII cells [35].

IPF and aging share multiple overlapping mechanisms, including the senescence of fibroblasts and resident stem cells. In the adult lung, resident stem cells, such as ATII cells, are essential for tissue repair. However, in susceptible individuals, chronic exposure to environmental insults leads to a shift from alveolar regeneration to fibroblast proliferation, ultimately promoting fibrosis [32,99].

ATII cell senescence is associated with a deficiency of mitochondrial sirtuin 3 (SIRT3), which contributes to increased myofibroblast proliferation. Extracellular vesicles facilitate cross-talk between fibroblasts and epithelial cells, further accelerating senescence in IPF [100,101]. During aging, ATII cells exhibit high levels of SASP, characterized by the production of pro-inflammatory factors such as plasminogen activator inhibitor 1 (PAI-1), tumor necrosis factor (TNF), endothelin-1, and platelet-derived growth factor (PDGF) [102]. Overexpression of PAI-1 is a significant driver of ATII cell senescence, acting in a self-perpetuating manner in conjunction with transforming growth factor-beta (TGF-beta) [103]. ER stress plays a central role in the inflammatory response associated with both viral infections and aging. In ATII cells, ER stress induces apoptosis, fibroblast activation, and epithelial–mesenchymal transition (EMT), culminating in fibrosis. These cells are particularly sensitive to ER stress due to inhibition of glucose-regulated protein (GRP) 78 and activation of TGF-beta [104,105]. Autophagy is generally a protective mechanism during ER stress, responsible for eliminating harmful cellular components. However, insufficient autophagy can promote cellular senescence, EMT, myofibroblast proliferation, and fibrosis [106]. Evidence suggests that autophagy in ATII cells is markedly deficient in IPF [35], and reduced levels of the key autophagy marker Beclin 1 have been noted in fibroblasts [107].

Aging and IPF are also linked to telomere shortening, which is observed in approximately 50% of IPF cases. In familial IPF, telomere-related genetic mutations account for one-third of cases. Mutations in telomerase components, such as telomerase reverse transcriptase (hTRT) or human telomerase RNA (hTR), are present in 10–15% of familial cases and 3% of sporadic cases. Telomere shortening contributes to immunodeficiency, particularly affecting T-cell function, which manifests clinically as recurrent cytomegalovirus infections. Additional extrapulmonary manifestations include varying degrees of bone marrow failure [108].

Although telomere shortening is an important component of IPF pathogenesis, it is insufficient by itself to induce lung disease. Secondary environmental insults, such as cigarette smoke or viral infections, are necessary to trigger IPF development. This explains why individuals with short telomeres develop diseases in high-turnover tissues, such as bone marrow, at a young age but do not present with IPF before the age of forty. Alder et al. analyzed the potential use of telomere length measurements and telomerase mutations in a hospital setting. Extremely short telomeres were observed in young adults with bone marrow failure and immunodeficiency. Additionally, short telomeres were also characteristic of pulmonary diseases such as emphysema and interstitial fibrosis [109]. Aging also impairs mitochondrial function, leading to increased ROS production and disruptions in mitochondrial DNA metabolism, which are commonly observed in IPF and result in accelerated cell death and further telomere attrition [110,111].

As individuals age, SIRT3 expression declines, contributing to increased mitochondrial ROS production and DNA damage. SIRT3 also plays an essential role in inhibiting TGF-beta1 activity and suppressing in vitro fibroblast and myofibroblast differentiation both in mice and humans [112].

Epigenetic changes have also been implicated in the pathogenesis of IPF. Dysregulation of microRNAs (miRNAs) plays a key role in the progression of the disease. For instance, profibrotic miRNAs such as miR-21 and miR-199a-5p are upregulated in IPF, while anti-fibrotic miRNAs such as miR-26a, miR-9-5p, miR-29, miR-200, and let-7d are downregulated. These changes lead to TGF-beta-mediated collagen synthesis and a shift in the balance between alveolar epithelial cells and fibroblasts, favoring fibrosis [113]. Additionally, alterations in DNA methylation and post-translational histone modifications contribute to IPF pathogenesis, as previously described by our group [114].

#### 4.5.4. Non-IPF Interstitial Lung Fibrosis

The molecular mechanisms of aging described earlier are likely implicated in the progression of fibrosing interstitial lung diseases (ILDs) beyond IPF. In systemic sclerosis (SSc), for example, these aging-related processes contribute to fibrosis in both the lungs (SSc-ILD) and the skin [115]. Yang et al. conducted a gene expression meta-analysis of lung tissues from 38 patients with SSc-ILD and 18 healthy controls, revealing a significant upregulation of senescence-associated markers (GDF15, COMP, CDKN2A) and pathways (p53). These findings highlight the increased expression of cellular senescence markers and pathways in SSc-ILD compared to healthy controls [116]. Additionally, patients with SSc-ILD exhibited shortened telomeres, with telomere lengths comparable to those seen in IPF patients [116]. Furthermore, SSc-ILD patients had lower levels of SIRT1 mRNA in their peripheral blood mononuclear cells (PBMCs) compared to SSc patients without pulmonary involvement [117]. In a study by Zhang et al., 22% of patients with fibrotic hypersensitivity pneumonitis (HP), unclassifiable ILD (uILD), and connective tissue disease-related ILD (CTD-ILD) were found to have telomeres shorter than the 10th percentile of normal [118]. Notably, patients with shorter telomeres had poorer survival outcomes when treated with immunosuppressive therapy [118]. However, another study reported that telomere length did not affect the rate of lung fibrosis progression in a large cohort of patients with IPF and non-IPF ILD [119]. A Mendelian randomization study identified an inverse correlation between shorter telomere length and the risk of several autoimmune diseases, including rheumatoid arthritis and sarcoidosis [120]. Other studies have shown that patients with sarcoidosis may have statistically shorter telomeres than healthy controls, although no definitive relationship has been established between telomere length and the severity of lung involvement or the presence of fibrotic sarcoidosis [121,122]. These findings suggest that while telomere shortening is a common feature in fibrotic ILDs, its role in disease progression may vary depending on the specific ILD subtype and other contributing factors.

## 5. Conclusions

The complete mechanisms underlying cellular senescence are not yet fully elucidated, but the link between aging and cellular senescence is well established. The aging of lung cells, as well as overall lung aging, must be understood within the broader context of organismal aging. It remains uncertain whether selectively targeting lung aging would offer significant therapeutic benefits in the near future. Aging-related diseases, including diabetes, atherosclerosis, heart failure, and pulmonary conditions, arise through both age-dependent and independent pathways. This complexity raises questions about whether addressing disease-driven cellular senescence is an optimal therapeutic approach, given that senescence may represent a protective response to environmental stressors.

Nevertheless, in the context of established diseases with overt clinical manifestations, therapies targeting cellular senescence may provide additional benefits. Approaches that address aging-related mechanisms in disease hold therapeutic promise, but predicting their outcomes is challenging due to the diverse etiologies and manifestations of aging-related diseases. Preventative strategies, especially those focusing on individuals at heightened risk for specific conditions, are likely to yield more immediate and tangible results. Consequently, research on risk factors and early interventions may offer faster progress in mitigating the impact of aging-related diseases.

## Figures and Tables

**Table 1:** Cellular senescence in physiology and pathology.

Effect	Process
Beneficial	Tumor supression
	Tissue regeneration
	Wound heeling
Detrimental	Tumor growth inspiration
	Acute inflammation
	Chronic inflammation
	Stem cells depression
Neutral	Natural aging
	Senescence self-perpetuation
